# Non-canonical RNA substrates of Drosha lack many of the conserved features found in primary microRNA stem-loops

**DOI:** 10.1038/s41598-024-57330-5

**Published:** 2024-03-20

**Authors:** Karen Gu, Lawrence Mok, Matthew J. Wakefield, Mark M. W. Chong

**Affiliations:** 1 St Vincent’s Institute of Medical Research, Fitzroy, VIC 3065 Australia; 2 Department of Medicine (St Vincent’s), University of Melbourne, Fitzroy, VIC 3065 Australia; 3 Walter and Eliza Hall Institute of Medical Research, Parkville, VIC 3052 Australia; 4 Department of Obstetrics and Gynaecology, University of Melbourne, Parkville, VIC 3010 Australia

**Keywords:** Computational biology and bioinformatics, Molecular biology

## Abstract

The RNase III enzyme Drosha has a central role in microRNA (miRNA) biogenesis, where it is required to release the stem-loop intermediate from primary (pri)-miRNA transcripts. However, it can also cleave stem-loops embedded within messenger (m)RNAs. This destabilizes the mRNA causing target gene repression and appears to occur primarily in stem cells. While pri-miRNA stem-loops have been extensively studied, such non-canonical substrates of Drosha have yet to be characterized in detail. In this study, we employed high-throughput sequencing to capture all polyA-tailed RNAs that are cleaved by Drosha in mouse embryonic stem cells (ESCs) and compared the features of non-canonical versus miRNA stem-loop substrates. mRNA substrates are less efficiently processed than miRNA stem-loops. Sequence and structural analyses revealed that these mRNA substrates are also less stable and more likely to fold into alternative structures than miRNA stem-loops. Moreover, they lack the sequence and structural motifs found in miRNA stem-loops that are required for precise cleavage. Notably, we discovered a non-canonical Drosha substrate that is cleaved in an inverse manner, which is a process that is normally inhibited by features in miRNA stem-loops. Our study thus provides valuable insights into the recognition of non-canonical targets by Drosha.

## Introduction

MicroRNAs (miRNAs) are small RNAs, ~ 22 nucleotides (nt) in length, that play a crucial role in regulating gene expression^1,2^. Mature miRNAs are generated from stem-loop structures embedded within primary (pri)-miRNA transcripts^3,4^. The biogenesis of most miRNAs requires two RNase III enzymes, Drosha and Dicer, that process precursors in a stepwise manner^3,4^. Drosha interacts with a dimer of the RNA-binding protein Dgcr8 to form the microprocessor complex^5–7^. In the nucleus, the microprocessor binds to and cleaves the stem-loop structure in the pri-miRNA, releasing a precursor (pre)-miRNA stem-loop intermediate from the flanking single-stranded RNA (ssRNA) segments^3,4^. The pre-miRNA is exported to the cytoplasm where it is further processed by Dicer. Dicer cleaves two helical turns (~ 21–22 bp) away from the Drosha cleavage sites, generating a miRNA duplex^3,4^. This miRNA duplex is then bound by an Argonaute protein and one (passenger) strand quickly dissociates while the other (guide) strand remains with the Argonaute to form the core of the miRNA-induced silence complex (miRISC)^8^. The mature miRNA guides the complex to target mRNAs that have complementarity to its seed region (2–7 nt from the 5’ end of the miRNA) to induce gene repression^2,3^.

Accurate processing of miRNA precursors is crucial because even a single nucleotide shift in the seed sequence can result in miRISC binding to a different set of mRNA targets. Because cleavage of the pre-miRNA by Dicer is at least partially dependent on the position of Drosha cleavage^9^, precise cleavage by Drosha is particularly critical. Pri-miRNAs have been shown to contain multiple sequence and structure motifs that ensure accurate Drosha cleavage.

Structurally, an optimal miRNA stem-loop has an extensively base-paired stem of ~ 35 ± 1 bp^10^ and a terminal loop that is larger than 10 nt^11^. Two strands of the stem are connected by a ssRNA apical loop and flanked by unstructured ssRNA segments at the base. The microprocessor binds to the dsRNA stem, with Dgcr8 positioned towards the apical loop and Drosha towards the base with the catalytic site located one helical turn (~ 11 bp) away from the basal junction^12,13^. Multiple motifs have been identified in miRNA stem-loops that are crucial for ensuring accurate Drosha cleavage. These motifs include the basal UG motif, the apical UGU/GUG (UGU) motif, the CNNC motif, the mGHG motif, and the midBMW motif^10,14–16^. These appear to be redundant as the presence of a single motif is sufficient to enable efficient and accurate cleavage by Drosha^10^. This explains why most natural pri-miRNAs have only a subset of motifs. In addition to the motifs present on the pri-miRNA stem-loop and its adjacent regions, recent studies have shown that several suboptimal stem-loops require an optimal stem-loop on the same transcript for efficient processing^17,18^.

Beyond pri-miRNAs, Drosha can also cleave mRNAs that harbor stem-loops resembling those in the pri-miRNAs^19^. However, the stem-loop products from this process rarely enter the miRNA biogenesis pathway. Instead, this cleavage primarily destabilizes the mRNA, leading to the repression of target gene expression. Direct Drosha cleavage-mediated gene repression has been demonstrated for the *Dgcr8*^20–22^, *Myl9*, *Todr1*^23^, *Ngn2*^24^, and *Nfib*^25^ mRNAs. Drosha cleavage of the *Dgcr8* mRNA has been observed in many cell types. Because Dgcr8 is part of the microprocessor, this cleavage is thought to function as an autoregulatory mechanism. Drosha cleavage of the other mRNAs has largely been observed in pluripotent stem cells and cleavage plays a crucial role in maintaining the pluripotency of these cells^19^.

High-throughput sequencing suggests that there may be dozens of mRNAs that are cleaved by Drosha^26,27^. While the features of pri-miRNA stem-loops have been extensively studied, the Drosha-targeted mRNAs have not been as well characterized. In this study, we employed high-throughput sequencing to capture Drosha-cleaved polyA RNA in mouse embryonic stem cells (ESCs) and characterized the features of these non-canonical stem-loop substrates of this enzyme. We show that while Drosha cleaved pri-miRNA stem-loops and mRNA stem-loops may appear similar at a superficial level, there are fundamental differences between these two groups of substrates.

## Results

### Capturing Drosha-cleaved polyA RNAs by Degradome-seq

**Figure 1:**
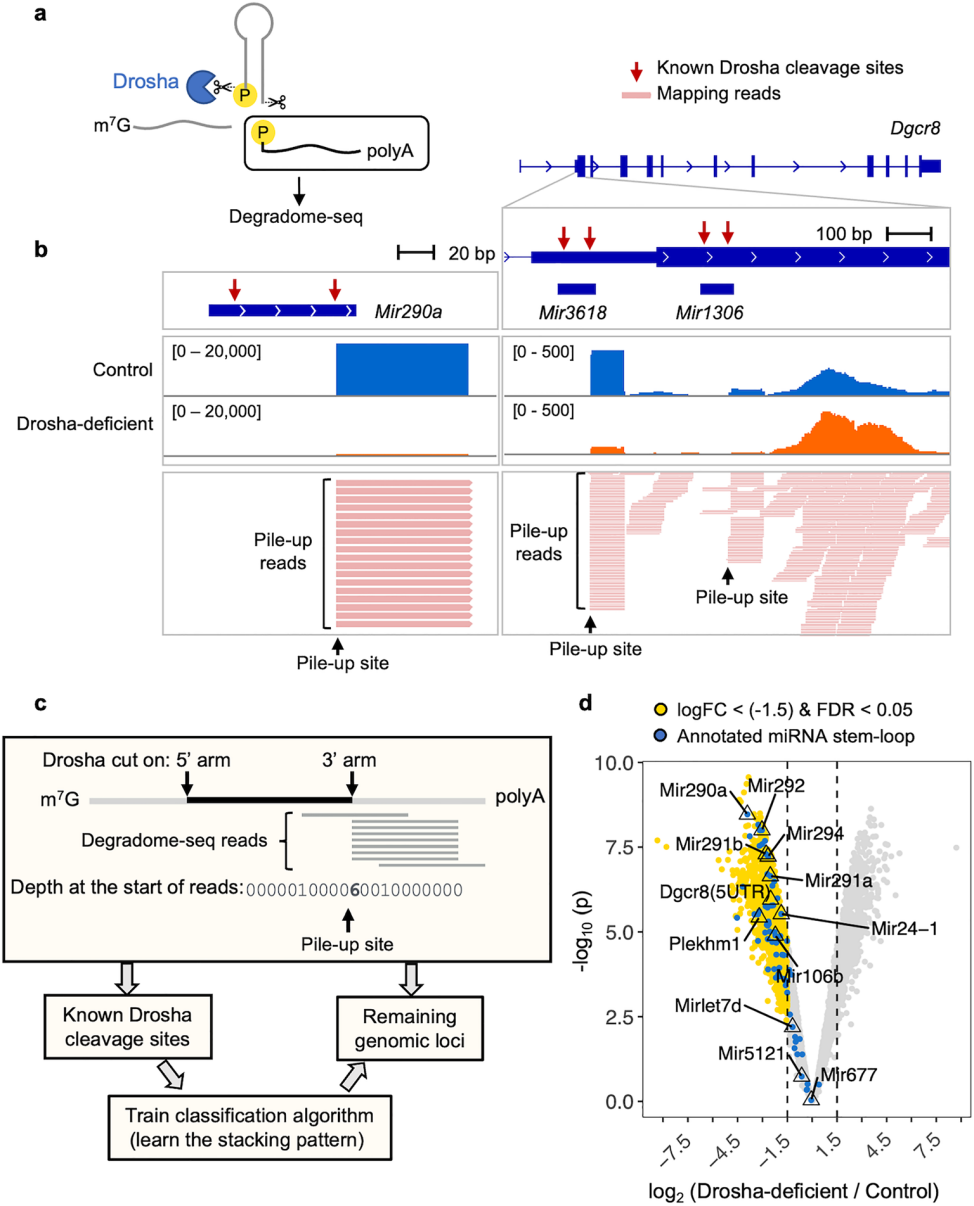
Analysis pipeline for identifying Drosha-dependent cleavage sites captured by Degradome-seq. (**a**) Degradome-seq captures polyA-tailed RNA with a 5’P, which is the hallmark of Drosha cleavage. (**b**) Example of Degradome-seq reads mapping to a miRNA gene (*Mir290a)* locus (left) and the stem-loops within the 5’UTR and CDS of *Dgcr8* (right). Both stem-loops in *Dgcr8* are also annotated as miRNAs. The gene/miRNA genomic positions are indicated in the top panel, with identified cleavage sites indicated by red arrows. Read depth for control and Drosha-deficient cells are shown in the next two tracks (representative of two replicates), with the data range for each track indicated in square brackets. The last track shows a collapsed view of aligned reads. (**c**) Schematic of the pipeline used to identify pile-up sites in Degradome-seq libraries. For each genomic locus, the depth at the start of the Degradome-seq reads was counted. The pile-up at annotated miRNA stem-loops (i.e. known Drosha cleavage sites) was used to train the classification algorithm for identifying the stacking pattern of Drosha-cleaved RNAs. The trained algorithm was then applied to the remaining genomic loci to identify those with similar stacking patterns. (**d**) Volcano plot of site pile-ups comparing Drosha-deficient and control ESCs. Sites that are significantly decreased (logFC < [− 1.5] and FDR < 0.05, 913 sites) in Drosha-deficient cells are indicated in yellow. Known cleavage sites on annotated miRNA stem-loops are indicated in blue.

Drosha is known to cleave mRNA in mouse ESCs^26^. To characterize the non-canonical RNAs that are directly cleaved by Drosha in mouse ESCs, we employed Degradome sequencing (Degradome-seq) to capture polyA-tailed RNAs with a 5′ monophosphate (5′P), a hallmark of Drosha-mediated cleavage (Fig. 1a). The Degradome-seq libraries were constructed essentially as described by Karginov et al.^26^ (Supplementary Fig. S1). PolyA RNA was first extracted, and an RNA linker was ligated to RNAs with a 5’P. The RNAs were then reverse transcribed with a random hexamer attached to a second (DNA) reverse linker sequence. PCR with primers against the 5′ and reverse linkers amplified fragments that have been endonucleolytically cleaved. To determine which cleavage sites are dependent on Drosha, we compared the sites between Drosha-deficient and control ESCs. These cells harbor a LoxP-flanked *Drosha*^*fl/fl*^ allele and CreERT2 knocked into the *Rosa26* locus^28^. Addition of 4-hydroxytamoxifen ablated Drosha expression and consequently the expression of canonical miRNAs, like miR-16 and miR-191, while the expression of the non-canonical miRNAs that are independent of Drosha, like miR-320 and miR-484^29^, were unaffected (Supplementary Fig. S2a and b). Two independent libraries were generated from both Drosha-deficient and control cells, each resulting in ~ 20 million reads mapping uniquely to the mouse reference genome GRCm38 (mm10; Supplementary Table S1).

We first assessed the quality of our Degradome-seq libraries. Even though the libraries were polyA enriched, the reads that mapped to the protein-coding genes do not skew towards the polyA tail, indicating that the libraries were not position-biased (Supplementary Fig. S2c). The depth of the reads was highly correlated between the two replicates (Supplementary Fig. S2d). When comparing control and Drosha-deficient libraries, the coverage of miRNA genes was decreased (Supplementary Fig. S2c–e), indicating that our libraries captured cleavage targets that are dependent on Drosha.

We next examined the reads that map to annotated canonical miRNAs loci. As expected, these reads exhibited a homogenous 5′ stack precisely at the Drosha cleavage site on the 3′ arm of the stem-loop (Fig. 1b, left panel). This detection of the 3′ cleavage site is expected because this protocol captures polyA-tailed RNA fragments (Fig. 1a). These stacked reads are referred to as “pile-up reads”, with the homogenous 5′ end referred to as the “pile-up site”. The depth of the pile-up site at the example *Mir290a* was reduced in Drosha-deficient ESCs, confirming that cleavage is Drosha-mediated (Fig. 1b, left panel).

An analogous stacking pattern was observed at other mRNA targets of Drosha. For example, Drosha-dependent pile-up sites were observed at the two annotated stem-loops in *Dgcr8,* one in the 5’ untranslated region (UTR) and one in the coding domain sequence (CDS; Fig. 1b, right panel). These results demonstrate the effectiveness of our Degradome-seq libraries for capturing Drosha cleavage sites in both pri-miRNAs and mRNA substrates.

Taking advantage of the clear stacking pattern observed in known substrates of Drosha, we developed a bioinformatics pipeline to systematically identify all Drosha-dependent cleavage events (Fig. 1c). We first counted the depth at the start of reads for each locus. Drosha cleavage sites mapping to miRNAs (as listed in MirGeneDB^57^) were used to train the classification algorithm, allowing it to learn the typical stacking pattern associated with Drosha cleavage. The successfully trained algorithm was then applied to the remaining loci to assess whether they exhibit a similar stacking pattern. This method identified 3632 pile-up sites in control cells, with 913 of these significantly decreased in Drosha-deficient cells (including pri-miRNAs; Fig. 1d; Supplementary Table S2). The pipeline successfully captured the cleavage of canonical pri-miRNAs, such as the *Mir290* ~ *295* family, while excluding Drosha-independent pri-miRNAs, such as *Mir5099* and *Mir677*^30^. Furthermore, Drosha-dependency was validated for selected target by qRT-PCR (Supplementary Fig. S3). These findings indicate that our pipeline successfully identifies Drosha-dependent cleavage of polyA RNAs in ESCs.

### Most of the Drosha-dependent cleavage sites are miRNA-independent

A Drosha-dependent pile-up site was identified in the *Plekhm1* mRNA (Fig. 1d). The cleavage of *Plekhm1* has previously been shown to be a miR-106b-5p-guided and is mediated by the slicer activity of Argonaute2^26^. This also leaves a 5’P on the cleaved RNA (Supplementary Fig. S4). Drosha is required for miR-106b biogenesis, and thus loss of Drosha affects *Plekhm1* cleavage indirectly.

To determine if other identified pile-up sites are also miRNA-dependent, we performed Degradome-seq on Dicer-deficient ESCs, in which the biogenesis of miRNAs will also be disrupted. Loss of miRNA expression was confirmed following *Dicer1* gene inactivation (Supplementary Fig. S5a and b). We generated two independent replicate Degradome-seq libraries each from Dicer-deficient and control ESCs (Supplementary Table S1). The depth of the reads was highly correlated between the two replicates (Supplementary Fig. S5c). Following processing, we identified 4265 pile-up sites in the control cells, of which 115 were lost in Dicer-deficient cells (Supplementary Fig. S5d; Supplementary Table S3). Of these, only 31 sites were affected by both Drosha and Dicer deficiency, and presumably via loss of miRNAs (Supplementary Fig. S5e). This suggests that most Drosha-dependent RNA cleavage sites in ESCs are independent of Dicer or miRNA.

### Inferring the secondary structure of RNAs that are cleaved by Drosha

**Figure 2:**
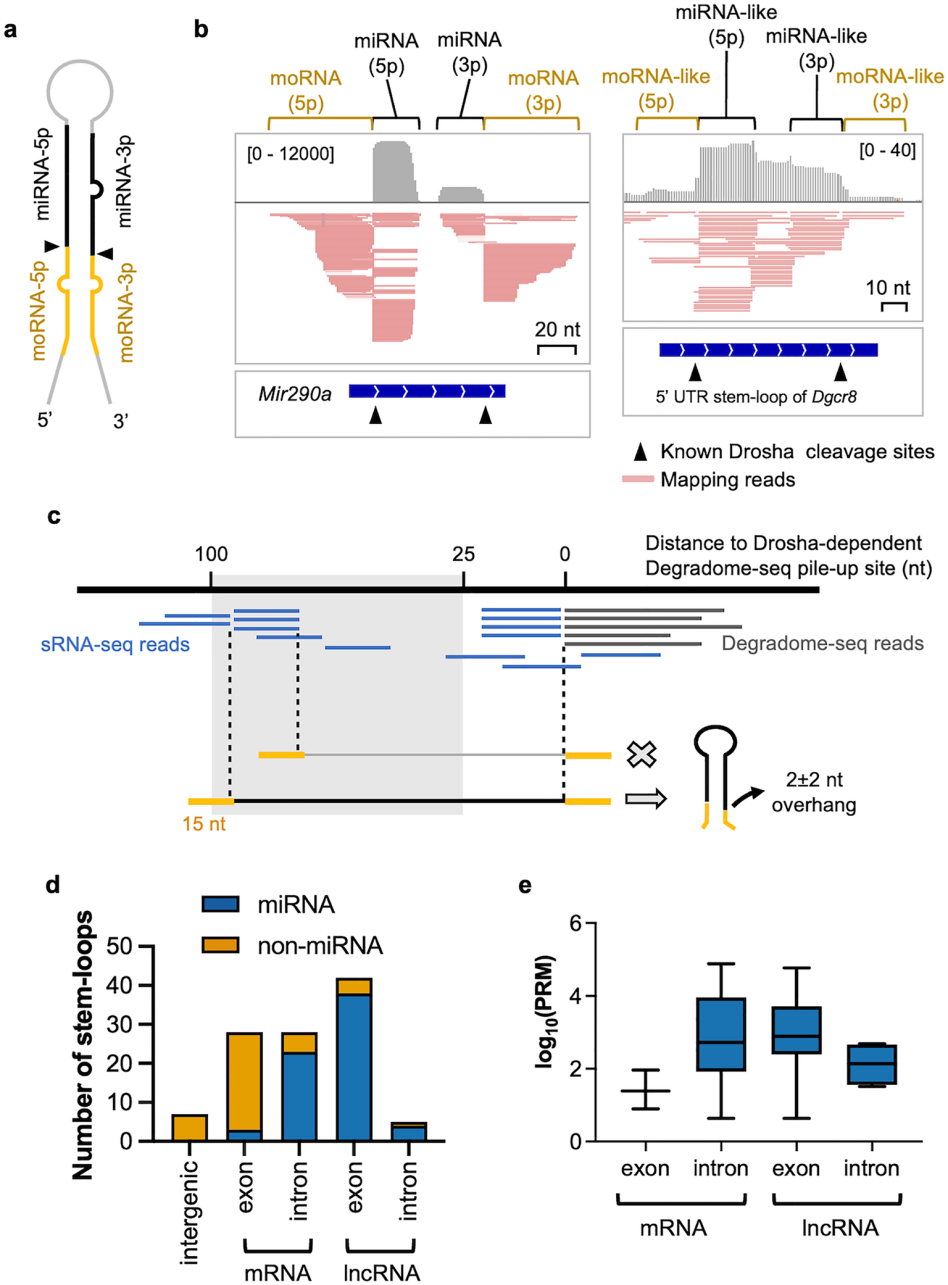
Identifying the boundaries of Drosha-cleaved stem-loops using miRNA and moRNA fragments from sRNA-seq. (**a**) Schematic representation of a miRNA stem-loop structure within the pri-miRNA transcript. Indicated are the positions from where the miRNA (black) and moRNA (yellow) fragments are derived. The Drosha cleavage sites are indicated by the black triangles. (**b**) sRNA-seq reads mapping to *Mir290a* and the 5’ UTR stem-loop of *Dgcr8*. The top panel shows the read depth followed by the aligned sRNA-seq reads in a collapsed view (data range indicated in square brackets). The bottom panel shows the relative position of the stem-loop of *Mir290a* and *Dgcr8*. (**c**) Strategy for identifying Drosha-cleaved stem-loops from sRNA-seq reads. Reads starting or ending between 25 and 100 nt from the Drosha-dependent Degradome-seq pile-up were collected. If the sRNA-seq reads stack at one of the termini and the depth of that terminus is greater than one read per million (RPM), it is considered a potential Drosha cleavage site for the opposite arm of the stem-loop. The RNA sequence between the terminus and the corresponding Degradome-seq pile-up site, flanked by a 15 nt sequence, was then used to predict the stem-loop structure. A stem-loop was considered a Drosha cleavage substrate if the terminus of the sRNA-seq reads and the Degradome-seq pile-up site formed a Drosha signature 3′ 2 ± 2 nt overhang. (**d**) Genomic locations of Drosha-cleaved stem-loops identified in ESCs. Annotated miRNA stem-loops are indicated in blue and non-miRNA stem-loops are indicated in orange. (**e**) The levels of annotated miRNAs derived from mRNAs and lncRNAs. The levels of each miRNA in mouse ESCs were obtained from mirGeneDB^57^.

Drosha usually cleaves stem-loops at two opposing strands to leave an RNase III enzyme signature 3′ 2 nt overhang^31^. To understand the nature of Drosha substrates, we first needed to identify the paired cleavage sites, i.e., the precise position of the cleavage on both the 5’ and 3’ arms of a putative stem-loop structure. Degradome-seq usually reveals the cleavage site on the 3’ arm, but not on the 5’ arm. To identify the 5’ cleavage site, we employed small (s)RNA-seq libraries, which capture miRNAs and other by-products of Drosha cleavage, such as miRNA-offset miRNAs (moRNAs) and other sRNAs. It has previously been shown that moRNAs are derived from the sequence immediately adjacent to the mature miRNA, with one end corresponding to the Drosha cleavage site and the other is thought to be processed by non-specific exonucleases^32^ (Fig. 2a). We reasoned that Drosha cleavage of non-miRNA stem-loops would also result in similar by-products that are captured by sRNA-seq.

We retrieved 38 sRNA-seq libraries of ESCs from the NCBI GEO database (Supplementary Table S4). Low-quality reads were first removed from each library, then the libraries were collapsed into unique reads and aligned to the mouse reference genome. Collapsing reads increased the representation of moRNAs and other low-frequency reads in the library to allow for easier identification. We confirmed that moRNAs do indeed map to miRNA loci immediately adjacent to the mature miRNA sequences, with the 3’ end of the moRNA-5p and the 5’ end of the moRNA-3p corresponding to the Drosha cleavage sites on the 5’ and 3’ arms of the pri-miRNA stem-loop (Fig. 2b). The distal termini of moRNAs were of varying lengths. This is different from the site-specific endonucleolytic cleavage signature and may indicate exonuclease-mediated degradation. Similar sRNAs were found adjacent to the Drosha-meditate cleavage site in the stem-loop at the *Dgcr8* mRNA and other non-miRNA Drosha cleavage targets (Fig. 2b and Supplementary Fig. S6). Here were refer to these as “miRNA-like” and “moRNA-like” sRNAs. Such by-product sRNAs appear to be common to Drosha substrates and not unique to miRNA precursors.

An algorithm was developed to utilize these moRNA-like sRNA-seq reads to determine the position of the cleavage site on the opposite arm of Degradome-seq pile-up sites in Drosha targets (Fig. 2c and Supplementary Fig. S7a). For this, we collected the sRNA-seq reads that started or ended between 25 and 100 nt from the Degradome-seq site, which corresponds to the size of known pre-miRNA stem-loops (Fig. 2c). Drosha can also produce a single cleavage on the 5p arm^33^. In this situation, the stem-loop and the sRNAs generated from the stem-loop would be downstream of the Degradome-seq pile-up site (Supplementary Fig. S7a). Therefore, the sRNA reads that are 25–100 nt downstream of the Degradome-seq site were also collected (Supplementary Fig. S7b). Of the Drosha-dependent sites identified by our Degradome-seq of ESCs, 801 exhibited this sRNA read pattern. If the sRNA-seq reads stacked at one of the termini and the depth of that terminus was greater than one read-per-million (RPM), it was regarded as a potential site of Drosha cleavage. The secondary structure of the sequence between the sRNA terminus and the corresponding Degradome-seq pile-up site, flanked by a 15 nt segment, was then predicted (Fig. 2c and Supplementary Fig. S7b). The flanking sequences were included to capture the entire putative stem-loop structure. A sequence that is predicted to fold into a stem-loop, with termini that formed an RNase III signature 3′ 2 nt overhang, was considered a cleavage target of Drosha (Fig. 2c and Supplementary Fig. S6b). This correctly identified the 5’ and 3’ Drosha-mediated cleavage sites in 68 miRNA stem-loops out of the 68 miRNAs that are highly expressed in ESCs, demonstrating the effectiveness of the algorithm. Using the same method, we were then able to reassemble 42 non-miRNA stem-loops that were cleaved by Drosha in ESCs (Supplementary Table S5).

The Drosha cleavage targets identified in this study largely overlapped with those captured in a previous analysis of mouse ESCs^26^ (Supplementary Fig. S8). This further supports the authenticity of Drosha cleavage targets identified. That being said, our study identified many more Drosha cleavage targets, which can be attributed to considerably deeper sequence depth. However, we found minimal overlap in Drosha cleavage targets reported for HEK293T and Hela cells^27^. This discrepancy is likely to be due to differences in the transcriptomes between mouse ESCs and immortal human cell lines. Moreover, many of Drosha targets with demonstrated biological function have been shown to be cell-type specific^23–25^.

Long non-coding (lnc)RNAs are a class of RNA that are longer than 200 nt. They usually possess a 5′ m^7^G cap and a 3′ polyA tail, but they do not encode functional proteins^34^. Many miRNA stem-loops are mapped to lncRNA genes, and the level of mature miRNAs derived from these transcripts is relatively high (Fig. 2d and e). Thus, these lncRNA are actually pri-miRNAs. One-third of the miRNA stem-loops were located in mRNA introns (Fig. 2d), which is consistent with previous studies^35^. Only three annotated miRNA stem-loops were located within an exon of a mRNA and these miRNAs tended to be expressed at low levels (Fig. 2e). Given the low level of mature miRNAs, these exon-located stem-loops are unlikely to be functional miRNA precursors. In contrast, most of the Drosha-targeted non-miRNA stem-loops were located in the exon of mRNAs (Fig. 2d).

### Drosha-cleaved non-miRNA stem-loops are less thermodynamically stable than miRNA precursor stem-loops

Drosha cleavage of exonic stem-loops is expected to destabilize the mRNA^22,23,25^. Stem-loops are one of the most common RNA structures^36^, and yet Drosha cleaves only some stem-loops but not others. We thus sought to understand the nature of the stem-loops that are recognized and cleaved by Drosha. For this, we systematically characterized and compared the features of miRNA and mRNA stem-loop targets of Drosha. If more than one alternative Drosha cleavage sites were identified for a stem-loop, the site with the highest number of sRNA-seq reads was selected for analysis.

**Figure 3:**
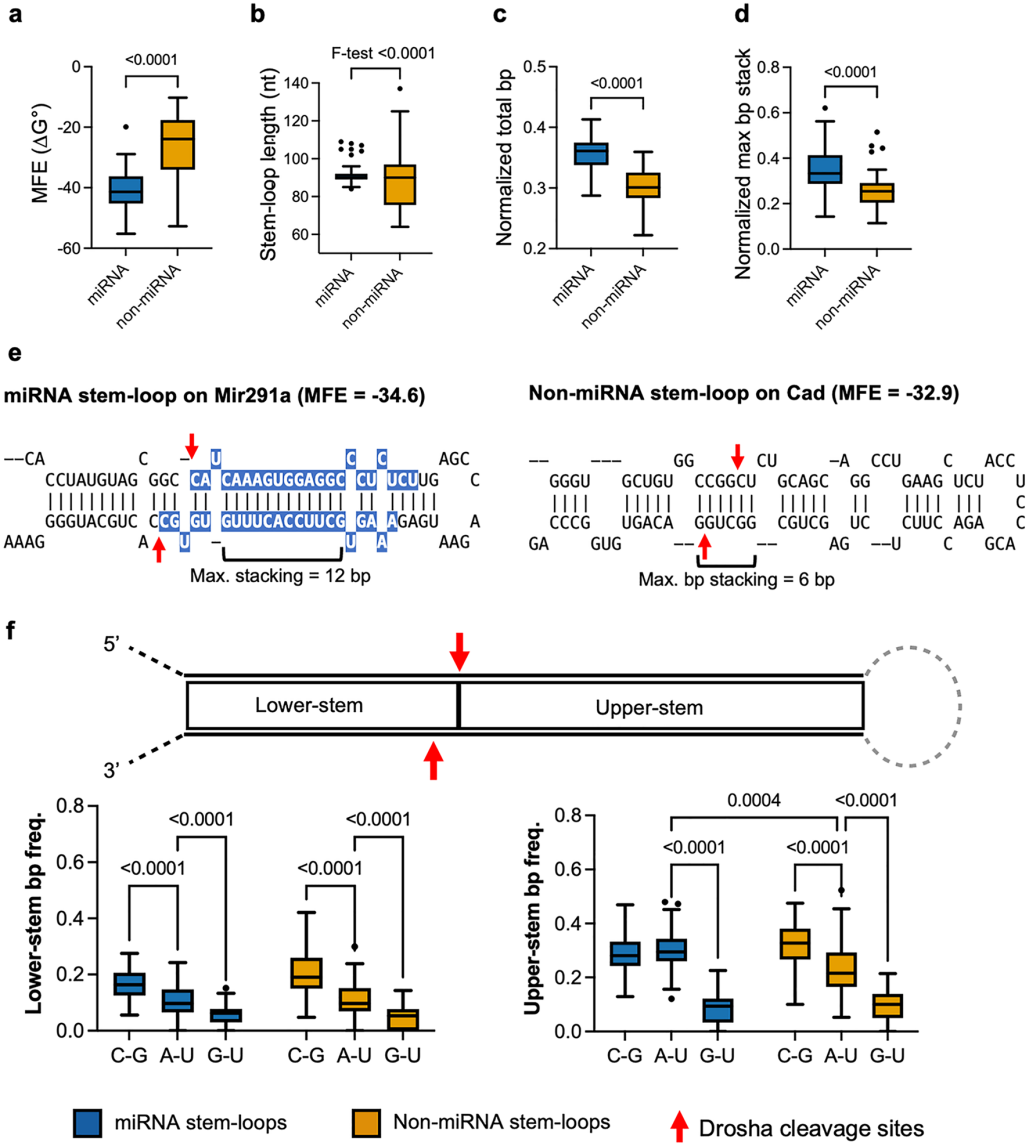
The thermodynamic properties of miRNA and non-miRNA stem-loops. (**a**,**b,c,d**) Comparison of the MFEs, stem-loop length, base pairing frequency and maximum stacking of base pairing frequency between Drosha-cleaved annotated miRNA and non-miRNA stem-loops identified in ECSs. Statistical testing: Two-tailed Welch’s *t-*test. *P*-values are indicated above the box. (**e**) The stem-loop in *Mir291a* and *Cad* are shown here to illustrate the typical difference between the miRNA and non-miRNA stem-loops. The miRNA sequences are highlighted in blue. The Drosha cleavage sites identified in ESCs are indicated by red arrows. (**e**,**f**) Comparison of the base pair composition of lower- and upper-stem of miRNA and non-miRNA stem-loops. The base pair frequency is normalized to the total number of base pairs of the stem. P-values are indicated above the box (two-way ANOVA). For **a**,**b,c,d** and **f**, the box represents the interquartile range (IQR), the whiskers extend to the most extreme data points within 1.5 times the IQR, and outliers are shown as individual points in boxplots. The median is represented by the line within the box.

We found that non-miRNA stem-loops had a significantly higher minimum free energy (MFE) compared to miRNA stem-loops, indicating that they were less thermodynamically stable (Fig. 3a). The stability can be affected by several properties, including RNA length, base pairing availability, and base pairing composition. We found that non-miRNA stem-loops are slightly shorter but had a significantly higher variance in length compared to the miRNA stem-loops (Fig. 3b). In addition, miRNA stem-loops had a higher base pairing frequency and a longer base pairing stacking (Fig. 3c-e). These results suggest that base pairing plays a significant role in the stability of miRNA stem-loops.

The composition of base pairing appeared to have less of an impact. Both miRNA and non-miRNA stem-loops exhibited similarly high G-C base pairing and low A-U and G-U base pairing in the lower-stem (Fig. 3f, left panel). However, only non-miRNA stem-loops exhibited a preference for high G-C base pairing in the upper-stem whereas there was a similar frequency of A-U and G-C base pairing in the upper-stem of miRNA stem-loops (Fig. 3f, right panel). Mature miRNAs originate from the upper-stem. This similar A-U and G-C usage in the upper-stem of miRNA stem-loops likely relates to the requirement for sequence diversity in miRNAs. Therefore, the stability of miRNA stem-loops is primarily achieved through extensive base pairing, while non-miRNA stem-loop relies mainly on the G-C base pairing.

### Non-miRNA stem-loops display more alternative structure

**Figure 4:**
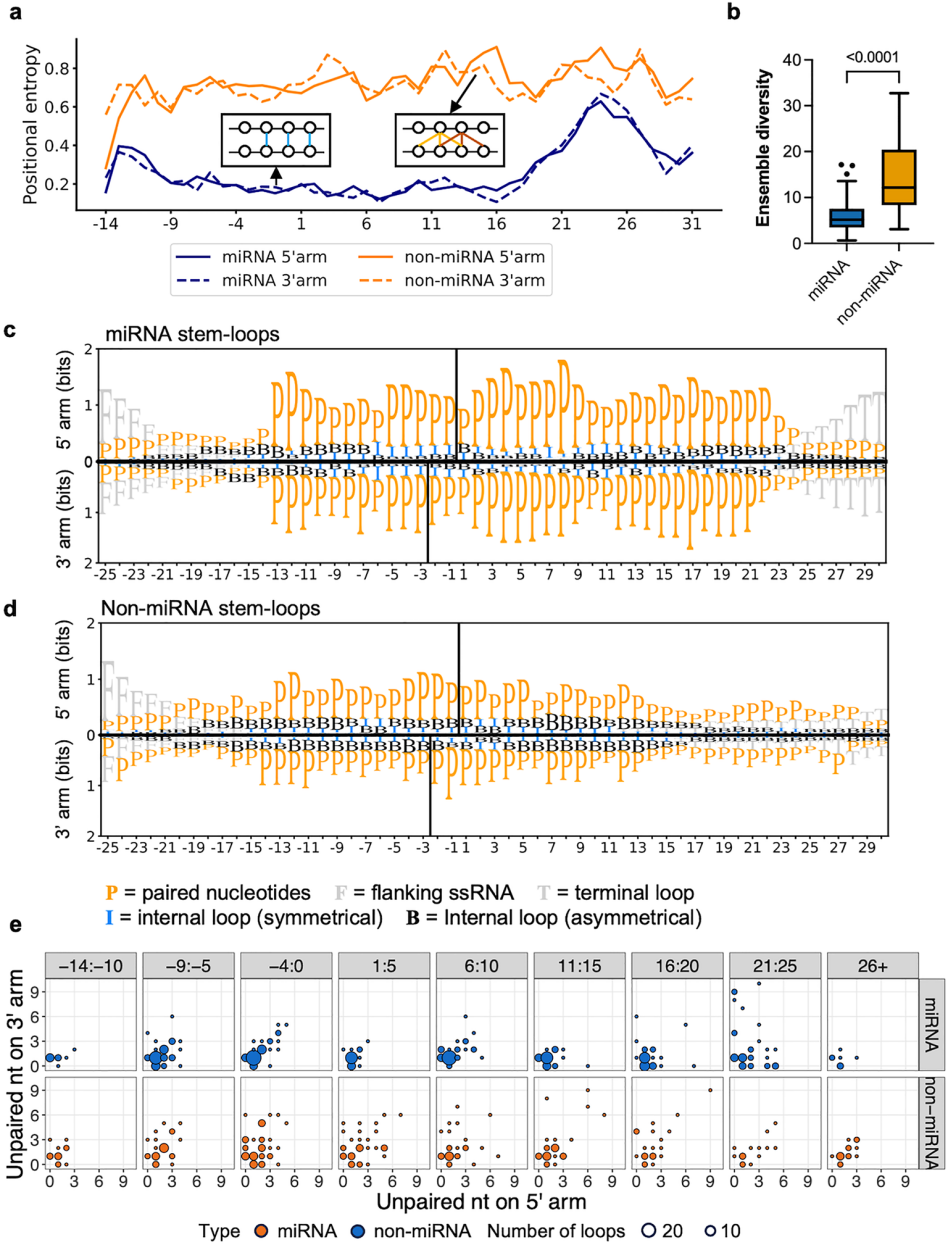
The structural features of miRNA and non-miRNA stem-loops. (**a**) Comparing the entropy of Drosha-cleaved annotated miRNA (blue) and non-miRNA (orange) stem-loops identified in ESCs. The 5’ and 3’ arms are plotted in solid and dashed lines, respectively. The x-axis shows the relative position of the stem-loop. Position 1 is the first nucleotide in the upper-stem. (**b**) Comparison of the ensemble diversity of miRNA and non-miRNA stem-loops. The box represents the IQR, the whiskers extend to the most extreme data points within 1.5 times the IQR, and outliers are shown as individual points. The median is represented by the line within the box. P-values are indicated above the box (independent Welch’s *t-*test, two-tailed). (**c**,**d**) Information bits plot comparing the structure of Drosha-cleaved (**c**) annotated miRNA and (**d**) non-miRNA stem-loops identified in ESCs. The 5′ arm is shown in the top panels of (**c**) and (**d**), while the 3’ arm is shown in the bottom panels. The position of the Drosha cleavage sites is indicated by a solid line. The x-axis indicates the relative position within the stem-loop, with Drosha cleavage site located between -1 and 1. P = paired nucleotides; F = flanking ssRNA; I = internal loop (symmetrical); B = Internal loop (asymmetrical); T = terminal loop. (**e**) Balloon plot of the size of internal loops within the stem in each 5 bp window. The size of the internal loop is indicated by the number of unpaired nt on the 5′ arm and 3′ arm. The position of the internal loop is indicated by the first unpaired nt that is closest to the Drosha cleavage site on the 5′ arm. The miRNA stem-loop is shown in the top panel in blue, and the non-miRNA stem-loops are shown in the bottom panel in orange. The size of the balloon corresponds to the number of internal loops that size in that window. Position 1 is the first nucleotide in the upper-stem.

An analysis of positional base pairing entropy shows that miRNA stem-loops have low entropy in the stem region, with a rise in entropy in the ssRNA region, including the unstructured flanking region and terminal loop region (Fig. 4a). These suggest that the stem of miRNA stem-loops is unlikely to form alternative base pairing, which likely ensures precise cleavage by Drosha and therefore the sequence of the mature miRNA that is eventually produced. In contrast, the entropy for non-miRNA stem-loops was consistently high, implying the presence of numerous alternative base pairing possibilities (Fig. 4a). This is further supported by the high ensemble diversity of non-miRNA stem-loops, which reflects the diversity of secondary structures that a non-miRNA stem-loop can adopt (Fig. 4b).

### Structural differences between miRNA and non-miRNA stem-loops

We next compared the fine structure of the stem-loops. Twenty-five nt of the sequence flanking the Drosha-cleaved stem-loop structure was included for these analyses. In agreement with the established understanding of the ideal length of miRNA stem-loops^10^, the miRNA stem-loops identified were found to be extensively base-paired between positions -13 and 22, creating a dsRNA stem of ~ 35 ± 1 bp in length (Fig. 4c). In contrast, non-miRNA stem-loops were extensively base-paired only between positions -13 and 13, resulting in a stem that is on average only ~ 26 bp in length (Fig. 4d). In addition, miRNA stem-loops have a conserved terminal loop region starting from position 25, while such a region was absent from the non-miRNA stem-loops. This is likely due to the variable stem length of non-miRNA substrates. Instead, a “weak” terminal loop can be observed starting from position 17 (Fig. 4d), suggesting that some non-miRNA stem-loop may have large terminal loops.

Non-miRNA stem-loops also displayed more asymmetrical internal loops compared to miRNA stem-loops. The miRNA stem-loops displayed small symmetrical internal loops in the lower-stem and the middle of the upper-stem (Fig. 4c and e). The internal loops at these positions have previously been shown to affect Drosha processing^10,15,33^. Large asymmetrical internal loops were mainly observed in the region 21 bp and above. These large asymmetrical internal loops serve as terminal loop in some miRNAs, such as in Mirlet7b (Supplementary Fig. S9). In contrast, numerous mismatches were observed in non-miRNA stem-loops, most of which formed asymmetrical internal-loops (Fig. 4d and e).

Despite the numerous structural differences between miRNA and non-miRNA stem-loop, their basal junctions were strikingly similar. A sharp decrease in pairing was observed at position -13 in both types of stem-loops (Fig. 4c and d), suggesting that a clear ssRNA-dsRNA junction is a crucial feature for Drosha cleavage. Similar to miRNA stem-loops, Drosha appears to bind to this ssRNA-dsRNA junction in non-miRNA substrates to cleave ~ 13 bp away from the junction.

### Non-miRNA stem-loop lacks canonical sequence motifs

**Figure 5:**
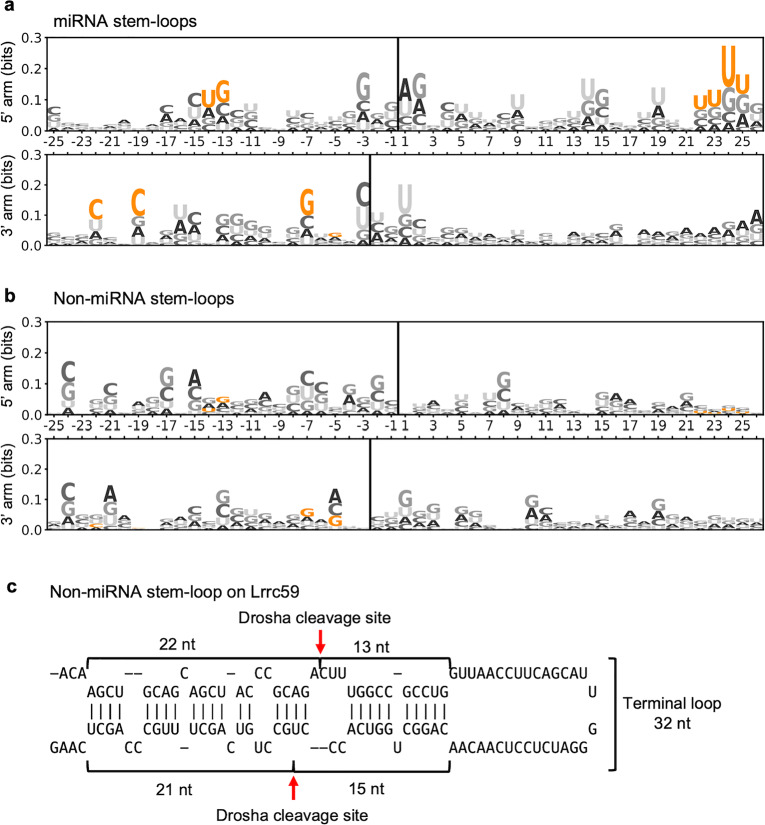
Non-miRNA stem-loops lack known miRNA sequence motifs. Logo of the sequence of Drosha-cleaved (**a**) annotated miRNA and (**b**) non-miRNA stem-loops identified in ESCs. The 5′ arm is shown in the top panels of (**a**) and (**b**), while the 3’ arm is shown in the bottom panels. The position of the Drosha cleavage site is indicated by a solid line. The x-axis indicates the relative position of the stem-loop. The y-axis shows the information bit of the sequence. Known miRNA motifs that are important for Drosha cleavage are highlighted in orange. (**c**) The non-miRNA stem-loop in *Lrrc59* is likely inversely cleaved due to its large terminal loop and lack of sequence motif. The Drosha cleavage sites are indicated by the red arrows.

Several conserved sequence motifs have been identified in miRNA stem-loop precursors and these have been shown to ensure efficient and accurate binding and processing by Drosha. These motifs include the basal UG motif, the apical UGU motif, the CNNC motif and the mGHG motif^10,14^. All sequence motifs were found in the Drosha processed miRNA stem-loops identified in ESCs (Fig. 5a). The apical UGU motif was found to be the most common motif in the miRNAs expressed in ESCs. In contrast, none of these miRNA motifs were detected in non-miRNA stem-loops (Fig. 5b).

Sequence motifs are important for the proper orientation of Drosha when it binds to the stem-loops. Without these motifs, Drosha can bind to the apical junction and cleave the stem-loop inversely^37^. While this phenomenon has been demonstrated using pri-miRNA variants in vitro with processing assays, whether it occurs in vivo has been unclear*.* Our analysis revealed a stem-loop in *Lrrc59* that is inversely cleaved by Drosha in ESCs. This stem-loop is characterized by an unusually large terminal loop of 32 nt. The Drosha cleavage site is ~ 13 bp away from the apical junction and has no clear basal junction (Fig. 5c), suggesting that Drosha binds to the apical junction and cleaves the stem-loop in an inverse manner. Together, these findings indicate that Drosha recognizes and processes miRNA and non-miRNA stem-loops differently.

### Drosha cleavage does not necessarily repress gene expression

While Drosha cleavage of transcripts can cause the significant destabilization and downregulation of transcript levels, we found this is not the case for many of the identified Drosha cleavage targets in our study. Differential gene expression analysis revealed that 1088 genes were significantly upregulated, and 375 genes were significantly downregulated (|logFC|> 1.5 and FDR < 0.05) in Drosha-depleted ESCs (Supplementary Fig. S10 and Supplementary Table S6). Except for Dgcr8, the cleavage of non-miRNA stem-loops results in only moderate to little reduction in transcript levels. This observation suggests that the cleavage of non-miRNA stem-loops might be either inefficient due to the absence of features that facilitate Drosha processing, or Drosha interaction serves a function other than destabilization for these transcripts.

## Discussion

Drosha cleaves many non-miRNA stem-loops in mouse ESCs. Our analysis of these non-canonical stem-loop substrates revealed fundamental differences between these and miRNA stem-loops. Specifically, we found that non-miRNA stem-loops are less thermodynamically stable and more likely to fold into alternative structures. Moreover, they lack the sequence and structural motifs normally found in miRNA stem-loops that ensure Drosha cleavage at single nucleotide precision. Consequently, Drosha can cleave these non-canonical substrates at positions that are typically inhibited in miRNA stem-loops.

Pri-miRNA stem-loops are typically thermodynamically stable and unlikely to fold into alternative structures. Many non-coding RNAs adopt specific secondary structures to carry out their functions, such as the clover-shaped transfer-RNAs. These RNAs have evolved over time, leading to exceptional thermodynamic stability and low structural diversity, which facilitate their processing and functionality^38,39^. Similarly, the stem-loop structure of miRNA plays a crucial role in its biogenesis^3^. Thus, like other functional non-coding RNAs, miRNA precursors have a stable structure that enables their efficient and accurate processing by Drosha and Dicer to produce functional miRNAs. However, a stable stem-loop in mRNA would hinder other processes, such as the progression of ribosomes along an mRNA thereby impairing protein expression^40^. Therefore, a stable stem-loop would be undesirable in mRNAs, which could explain why non-miRNA stem-loops are generally less stable.

The structure and sequence motifs of miRNA stem-loops are important for accurate binding and processing by Drosha. Drosha needs to bind to the ssRNA-dsRNA junction at the base and cleave ~ 11 bp away to produce miRNA intermediates^12,13^. However, due to the symmetrical structure of a stem-loop (i.e. ssRNA-dsRNA-ssRNA), Drosha can also bind to the apical junction, which could result in inverse cleavage of the stem, closer to the loop than to the base. To prevent this, several miRNA stem-loop sequences and structure motifs have been found in miRNA stem-loops to encourage binding of Drosha to basal junction and therefore inhibit inverse cleavage. This includes the UGU motif at the apical junction that interacts with Dgcr8, positioning Drosha towards the lower-stem^41^, while the CNNC motif interacts with Srsf3 to recruit Drosha to the lower-stem^14,42^. The basal UG and mGHG motifs also serve as Drosha-interacting motifs, enabling precise binding to the stem-loop^10,13^. Recently, a MidMW10 motif, located 10–12 nt away from the Drosha cleavage site in the upper-stem has also been shown to be essential for preventing inverse cleavage^15^. None of these structure and sequence motifs were found in non-miRNA stem-loops that are cleaved in ESCs. Consequently, inverse cleavage of non-miRNA substrates by Drosha, such as the stem-loop in *Lrrc59* that we described, is possible.

A difference between miRNA and non-miRNA stem-loops was anticipated because Drosha cleavage of mRNA stem-loops generally does not produce meaningful quantities of mature miRNAs. Consequently, many features required for precise miRNA production are unnecessary for non-miRNA stem-loop cleavage. However, the absence of Drosha recognition and processing features renders Drosha cleavage less efficient in repressing the expression of transcripts harboring non-miRNA stem-loops. This raises the question of why Drosha cleaves them at all. One possibility is that the cleavage serves a purpose other than destabilization, such as promoting alternative intron splicing. This has been demonstrated by Havens et al.^43^ and Lee et al^44^, where cleavage of a stem-loop promotes alternative splicing but has little impact on the gene expression level.

Even though the cleavage of most non-miRNA stem-loops is not efficient enough to repress gene expression in the ESCs, the possibility remains that *trans-* or *cis-*acting elements may facilitate Drosha-mediated cleavage of non-canonical stem-loop substrates to achieve spatiotemporally regulation of specific targets. It has been shown that post-transcriptional modification of target RNA can affect Drosha processing. N^6^-methyladenosine (m^6^A) on the RNA upstream of the stem-loop has been shown to recruit Drosha to the vicinity of pri-miRNA stem-loops and enhance Drosha processing efficiency^45^. Such modification may also be present upstream of non-miRNA stem-loops to ensure Drosha recruitment and to enhance cleavage efficiency. Additionally, the stability of stem-loops may be affected by ADAR enzyme-dependent A-I editing, thereby altering Drosha's processing efficiency on the stem-loop^46^.

Accessory proteins of the microprocessor may also affect non-canonical stem-loop processing. Although the minimal microprocessor complex of Drosha and Dgcr8 alone is sufficient to process pri-miRNA stem-loops, numerous accessory proteins that interact with the complex have been identified. These include the DEAD-box helicases (Ddx5) and tumor suppressor p53 that are required for Drosha-mediated processing of a subset of miRNAs^47^. Additionally, hnRNP TAR DNA-binding protein 43 (Tdp43) can interact with Drosha to increase its stability and promote processing^48^. Such accessory proteins may facilitate the processing of non-canonical miRNA stem-loops.

Furthermore, post-translational modifications can also regulate the microprocessor. Acetylation of lysine residues within the N-terminal of Drosha has been found to repress ubiquitin-mediated proteasome decay^49^. Deacetylation of Dgcr8 by histone deacetylase 1 (Hdac1) has been found to increase the affinity of Dgcr8 for a subset of pri-miRNA^50^. At least 23 phosphorylated amino acids have been found on Dgcr8. Phosphorylation appears to increase the stability of Dgcr8 without affecting its ability to interact with Drosha^51^. Similar post-translational modifications can regulate both protein–protein and protein-RNA interactions, thereby affecting Drosha’s processing efficiency on non-canonical stem-loop targets.

Our analysis is likely underestimating the number of possible Drosha targets. We focused on stem-loops where the two cleavage sites are at most 100 nt apart on the same RNA. Structural studies of Drosha^12,13^ and Drosha targets (Fig. 4c and d) suggest that the ssRNA-dsRNA junction is a crucial feature for successful Drosha cleavage. Such a ssRNA-dsRNA junction can also be present in longer stem-loops or between a pair of sense:antisense transcripts. It is possible that these might also be recognized and cleaved by Drosha but further studies are required to explore these possibilities.

Our study has provided a comprehensive analysis of stem-loops cleaved by Drosha in ESCs, revealing that the non-canonical stem-loop substrates of Drosha differ significantly from pri-miRNA stem-loops. Drosha cleavage-mediated gene repression has been shown to be critical for safeguarding the pluripotency of the stem cells^23–25^. Determining how this Drosha cleavage of non-canonical targets is achieved will therefore be critical for understanding the mechanisms regulating stem cell pluripotency, and the knowledge from this study provides a foundation for such future studies into the regulation of Drosha function.

## Materials and methods

### Generation of conditional Dicer ESCs

Conditional LoxP-flanked *Dicer*^*fl/fl*^ ESCs were derived from blastocysts obtained from *Dicer1*^*fl/fl*^ mice^52^. A time-mated female mouse was euthanized by CO_2_ asphyxiation 3 days after the vaginal plug was confirmed. The uterine horns were harvested, and blastocysts flushed out with PBS. Individual blastocysts were then deposited into wells containing Mitomycin C-inactivated mouse embryonic fibroblasts as feeder cells and cultured in KnockOut DMEM (Gibco) supplemented with 20% Knockout Serum Replacement (Gibco), non-essential amino acids (Gibco), 0.1 mM β2-mercaptoethanol and 10^3^ U/mL LIF (Peprotech). The wells were monitored daily for outgrowth from the blastocysts. Upon outgrowth, the well was trypsinized into a single cell suspension and transferred into a larger well for picking of individual colonies. This animal work was approved by the St Vincent’s Hospital Animal Ethics Committee. The experiments were performed in accordance with relevant guidelines and regulations under the Australian code for the care and use of animals for scientific purposes.

### Mouse ESCs culturing

Conditional LoxP-flanked *Drosha*^*fl/fl*^* Gt(ROSA)26Sor*^*CreERT2*^ ESCs have been described previously ^28^. All ESCs were cultured in KnockOut DMEM, supplemented with 10% heat-inactivated fetal bovine serum (GE Healthcare Life Sciences), 5% KnockOut Serum Replacement (Gibco), 1% sodium pyruvate (Gibco), 1% non-essential amino acids, 1% penicillin–streptomycin-glutamine (Gibco), 0.1 mM 2-mercaptoethanol and 10^3^ U/mL LIF on a Mitomycin C-inactivated mouse embryonic fibroblasts.

Deletion of the floxed Drosha allele was achieved by adding 100 nM 4-hydroxytamoxifen (Sigma-Aldrich) to the *Drosha*^*fl/fl*^* Gt(ROSA)26Sor*^*CreERT2*^ ESCs for 72 h. The medium was then replaced (without 4-hydroxytamoxifen) for a further 48 h to allow for depletion of Drosha-dependent miRNAs before analysis.

Deletion of the floxed Dicer allele was achieved by transducing the *Dicer*^*fl/fl*^ ESCs with a Cre-expressing retrovirus^53^. The virus also contained a GFP reporter that allowed for the sorting of transduced cells. GFP^+^ ESCs were sorted 3 days after transduction, then cultured for a further 2 days before analysis.

### Quantitative (q)RT-PCR

Total RNA was extracted from the cells using TRIsure (Bioline) following the manufacturer’s instructions. For measuring mRNA expression, 1 μg total RNA was reverse transcribed with 50 ng random hexamers using M-MuLV reverse transcriptase (NEB). 1/20th of the resulting cDNA was used and then analyzed by qRT-PCR using GoTaq qPCR master mix (Promega). The following primer pairs were used:

5′-GACGACGACAGCACCTGTT-3′ and 5′-GATAAATGCTGTGGCGG-ATT-3′ for Drosha;

5′-TCTGCAGGCTTTTACACACG-3′ and 5′-CAGCCAATGATGCAAA-GATG-3′ for Dicer;

5′-CACAGCTTCTTTGCAGCTCCTT-3′ and 5′-CGTCATCCATGGCGAAC-TG-3′ for β-actin.

Taqman miRNA assays (Thermo Fisher) were used to quantify the expression of mature miRNAs and U6 snRNA (control). For each target, 10 ng total RNA was reverse transcribed with its specific reverse transcription primer using 25 U Multiscribe reverse transcriptase. qRT-PCR then performed on 1/15th of the cDNA with Taqman universal PCR master mix and 1X Taqman miRNA assay mix (miR-16 assay ID: 000391; miR-191 assay ID: 002299; miR-320 assay ID: 002277; miR-484 assay ID: 001821; U6 snRNA assay ID: 001973).

### Degradome sequencing library construction

Degradome-seq libraries were constructed based on a previously described protocol^26^. In brief, polyA-tailed RNA was isolated from 75 μg of total RNA using the Dynabeads mRNA direct purification kit (Thermo Fisher) following the manufacturer’s instructions. An RNA linker (5′-CACGACGCUCUUCCGAUCU-3′) was ligated to the polyA RNA with T4 RNA ligase (Thermo Scientific) to capture RNAs with a 5’P, a hallmark of RNase III cleavage. The ligation products were then purified with the Dynabeads mRNA direct purification kit and reverse transcription was performed using Superscript III reverse transcriptase (Invitrogen) and random hexamer primer attached to reverse adaptor sequence (5′-AGACGTGTGCTCTTCCGATCNNNNNN-3′). The cDNA was cleared of RNA by treating it with RNase H (NEB). Half the cDNA was subjected to 2nd strand synthesis using Phusion high-fidelity DNA polymerase (NEB) with primers to the 5′ RNA linker and reverse adaptor (5′-ACACTCTTTCCCTACACGACGCTCTTCCGATCT-3′ and 5′-GTGACTGGAGTTCAGACGTGTGCTCTTCCGATC-3′) using the cycling conditions: 98 °C for 1 min, (98 °C for 30 s, 58 °C for 30 s, 72 °C for 1 min) × 7 cycles, and 72 °C for 5 min. The resulting cDNA was resolved on a Low Melting Point agarose gel (Scientifix) and cDNA corresponding to 200–400 bp were gel purified. This cDNA library was then PCR barcoded with PCR Primer 1.0 (5′-AATGATACGGCGACCACCGAGATCTACACTCTTTCCCTACACGACG-3′) and a sample-specific barcode primer (5′-CAAGCAGAAGACGGCATACGAGATNNNNNNGTGACTGGAGTTCAGACGTG-3’) using MyTaq Red Mix (Bioline) for 15 cycles. The resulting cDNA libraries were sequenced on the NextSeq 500 platform (Illumina) for 75 cycles in single-end high-output mode at the Australian Genome Research Facility.

### Gene-specific degradome qRT-PCR

PolyA-tailed RNA was isolated from 75 μg of total RNA using the Dynabeads mRNA direct purification kit following the manufacturer's instructions. An RNA linker (5′-CACGACGCUCUUCCGAUCU-3′) was ligated to the polyA RNA with T4 RNA ligase to capture RNAs with a 5′P. The ligation products were then purified with the Dynabeads mRNA direct purification kit and reverse transcription was performed using Superscript III reverse transcriptase and random hexamer primers.

One-twentieth of the resulting cDNA was pre-amplified for 20 cycles using 5 U Taq DNA polymerase (NEB), with 1 μM forward primer to the 5′ RNA linker (5′-ACTCTTTCCCTACACGACGC-3′) and 1 μM gene-specific outer reverse primers: 5′-TTCATGGGGCAGCACTTGGA-3′ for Dgcr8; 5′-TGGCCGAATCTGCTACTTCAC-3′ for Rcan3; and 5′-TCTGTCCGTCACCTTGCCTT-3′ for Chpf2. One-hundredth of resulting pre-amplified cDNA was then analyzed by qPT-PCR using GoTaq qPCR master mix with 0.5 μM forward primer to the 5′ RNA linker (5′-ACACTCTTTCCCTACACGACGCTCTTCCGATCT-3′) and 0.5 μM gene-specific inner reverse primers: 5′-AGGGACTCTCATATGTCTCCA-3′ for Dgcr8; 5′-CTCAGAGTGCACAGTCCAGC-3′ for Rcan3; and 5′-TGTTGGCCTGTTCCTGTTCA-3′ for Chpf2.

### Sequence processing and alignment of Degradome-seq libraries

Processing and alignment of Degradome-seq libraries were performed on the Galaxy Australia platform^54^. The raw reads were processed using the Trimmomatic program^55^ (version 0.38.0) to remove Illumina platform-specific adaptor sequences and low-quality bases (below 20 across 4 nt). The processed reads were then aligned to the mouse reference genome GRCm38 (mm10) using RNA STAR with default parameters^56^ (version 2.7.8a).

### Degradome-seq analysis pipeline

The depth of Degradome-seq reads at each locus was determined by counting the number of reads starting at that locus. The raw counts were normalized as the reads per million (RPM). The depth of read at the 5’ end was denoted by $C5$. For each genomic locus i, we generated a counting vector ${C}_{i}$ by

$${C}_{i}=C{5}_{i-10}, C{5}_{i-9}, C{5}_{i-8}, \dots , C{5}_{i}, C{5}_{i+1}, \dots , C{5}_{i+10}.$$

We retrieved a list of miRNAs from MirGeneDB^57^. The genomic loci immediately upstream of the 5' end of the 5' arm miRNA or downstream of the 3' end of the 3' arm miRNA were considered as known Drosha cleavage sites. The counting vector of these known Drosha cleavage sites was utilized as the positive training dataset. A set of 1000 loci was randomly selected from the rest of the genome as a negative training dataset. These positive and negative training datasets were used to train a generalized linear model using the *cv.glmnet* function in the R package *glmnet*^58^, with the following parameters: family = “binomial”, type.measure = “class”, nfolds = 10. The training error was accessed by sensitivity, specificity, and the area under the curve (AUC). The AUC was calculated using the R package *pROC*^59^ (version 1.18.0). The sensitivity and specificity were calculated using the formulas below:

$$Sensitivity= \frac{TP}{TP+FN} ,$$

$$Specificity= \frac{TN}{TN+FP} ,$$

where $TP$ is true positive, $FN$ is false negative, $TN$ is true negative, and $FP$ is false positive. We considered the model trained successfully if it had sensitivity > 0.9, specificity > 0.9, and AUC > 0.9. We then applied the model to the rest of the genomic loci to identify those with a similar stacking pattern (i.e., pile-up site) to the known pri-miRNAs. The step between randomly selecting 1000 negative samples and applying the model to the rest of the genomic loci was repeated until the datasets were classified 1000 times. A locus was considered a true pile-up site if it was classified as a pile-up site in more than 900 iterations in both control ESC replicates.

To determine whether a pile-up site was dependent on Drosha or Dicer, we performed differential pile-up analysis between Drosha deficient and control ESCs or Dicer deficient and control ESCs using the *edgeR* package^60^ (version 3.26.1). Raw read counts starting at each genomic locus were normalized by the trimmed mean of M values (TMM), and the dispersion was estimated by calling the function *estimateDisp()*. The testing of differential pile-ups was performed by calling functions *glmQLFit()* and *glmQLFTest()*. A genomic locus with a log_2_ fold change (log_2_FC) < (− 1.5) and false discovery rate (FDR) < 0.05 were considered significant.

### Sequence processing and alignment of small RNA sequencing libraries

Small RNA sequencing (sRNA-seq) libraries used in this study are retrieved from the NCBI Gene Expression Omnibus (GEO) database (Supplementary Table S1) and the processing and alignment of reads were performed on the Galaxy Australia platform^54^. The quality of the reads and the representation of the sequencing adapters were accessed by the FastQC program (version 0.72), and the reported adapter sequences were trimmed using cutadapt^61^ (version 1.16). The processed reads were collapsed into unique reads using an in-house. The unique reads were then aligned to the mouse reference genome GRCm38 (mm10) using RNA STAR with default parameters^56^ (version 2.7.8a).

### Identification of Drosha-cleaved stem-loops

Positions identified as Drosha-dependent cleavage sites in the Degradome-seq analysis were denoted as ${D}_{i}$. Reads starting or ending between ${D}_{i-100}$ and ${D}_{i-25}$ or between ${D}_{i+25}$ and ${D}_{i+100}$ were collected. If the sRNA-seq reads stack at one of the termini and the depth of that terminus is > 1 RPM (denoted as ${S}_{j}$), the RNA sequence between ${S}_{j-15}$ and ${D}_{i+15}$ or between ${D}_{i-15}$ and ${S}_{j+15}$ was used to predict the secondary structure using *RNAfold* program in the *ViennaRNA* package^62^ (version 2.4.17) with the following option: RNAfold -p -d2 –noLP –MEA. The 3′ overhang was calculated in the structure generated by *RNAfold*. The structure was considered an authentic Drosha-cleaved stem-loop if ${S}_{j}$ and ${D}_{i}$ form a 3′ 2 ± 2 nt overhang.

### Overlapping analysis

To obtain a list of Drosha cleavage targets captured in Degradome-seq libraries from Karginov et al.^26^, we reanalyzed the Degradome-seq dataset using the method developed in this study. The Drosha cleavage targets identified in HEK293T and HeLa cells^27^ were converted to mouse genes using “Mouse/Human Orthology with Phenotype Annotations” obtained from Mouse Genome Informatics (MGI). The identification of overlapping Drosha targets was performed using the *ggVennDiagram* package^63^.

### Differential gene expression analysis

Stranded RNA sequencing (RNA-seq) libraries were prepared using the TruSeq Stranded mRNA Sample Preparation Kit from 3 μg total RNA. The libraries were sequenced on the Illumina NovaSeq 6000, generating 20 million 100 bp single-end reads at Australian Genome Research Facility. The raw reads were aligned to the mouse reference genome GRCm38 (mm10) using RNA STAR with default parameters. The differential gene expression analysis was performed using *edgeR* package^60^. Genes with |log_2_FC|> 1.5 and FDR < 0.05 were considered to be significantly differentially expressed.

### Analysis of stem-loop thermodynamic properties

The length of the stem-loop was determined as the distance between ${D}_{i}$ and ${S}_{j}$ with a 15 nt flanking sequence. The base pairing frequency and maximum stacking of base pairing were normalized to the length of the stem-loop. The base pair composition was normalized to the total number of base pairs in the stem.

### Analysis of stem-loop structural diversity

The ensemble diversity of the stem-loop was obtained from the *RNAfold* output using the option described above. The positional entropy of the stem-loops was calculated using script *mountain.pl* in the *ViennaRNA* package^62^ (version 2.4.17).

### Information bits plot of structural features

The secondary structure between ${D}_{i}$ and ${S}_{j}$ with a 25 nt flanking sequence was predicted using the *RNAfold* program with the following option: RNAfold -p -d2 –noLP -C. The position of paired bases in secondary structure between ${D}_{i}$ and ${S}_{j}$ with a 15 nt flanking sequence was used as a constraint to inform the prediction. This ensured that paired bases remained paired when incorporated with longer flanking segments.

An in-house script was used to determine whether a position forms a base pair, an internal loop (symmetrical or asymmetrical), a terminal loop, or flanking segments. The resulting data was used to generate information bits plots with the Python package *Logomaker*^64^ (version 0.8).

### Sequence Logo

The sequence logo was generated using the Python package *Logomaker*^64^ (version 0.8) and the sequence between ${D}_{i}$ and ${S}_{j}$ with a 25 nt flanking sequence.

### Statistical analysis

Two-tailed *t*-tests were used to perform statistical analysis on the qPT-PCR data. If not indicated, two-tailed unequal variance *t*-tests (Welch’s *t*-test) were used to analyze the differences between miRNA and non-miRNA stem-loop features. Results were considered statistically significant at *p* < 0.05.

### Supplementary Information

Supplementary Figures.

Supplementary Tables.

## Data Availability

The data underlying this article are available in NCBI GEO and can be accessed with accession number GSE228299. All methods are reported in accordance with ARRIVE guidelines (https://arriveguidelines.org).
