# Supplementary material for: Non-canonical RNA substrates of Drosha lack many of the conserved features found in primary microRNA stem-loops
Source: Sci Rep. 2024 Mar 20;14:6713. doi: 10.1038/s41598-024-57330-5 (PMC10954719; doi:10.1038/s41598-024-57330-5)
Supplement: Supplementary file 1 — Supplementary Figures. [file 41598_2024_57330_MOESM1_ESM.pdf]

SUPPLEMENTARY INFORMATION

Non-canonical RNA substrates of Drosha lack many of the conserved features found in primary microRNA stem-loops

Karen Gu[1,2], Lawrence Mok[1], Matthew J. Wakefield[3,4] and Mark M.W. Chong[1,2,*]

[1] St Vincent's Institute of Medical Research, Fitzroy, Victoria, 3065, Australia

[2] Department of Medicine (St Vincent's), University of Melbourne, Fitzroy, Victoria, 3065, Australia

[3] Walter and Eliza Hall Institute of Medical Research, Parkville, Victoria, 3052, Australia

[4] Department of Obstetrics and Gynaecology, University of Melbourne, Parkville, Victoria, 3010, Australia

* To whom correspondence should be addressed: mchong@svi.edu.au

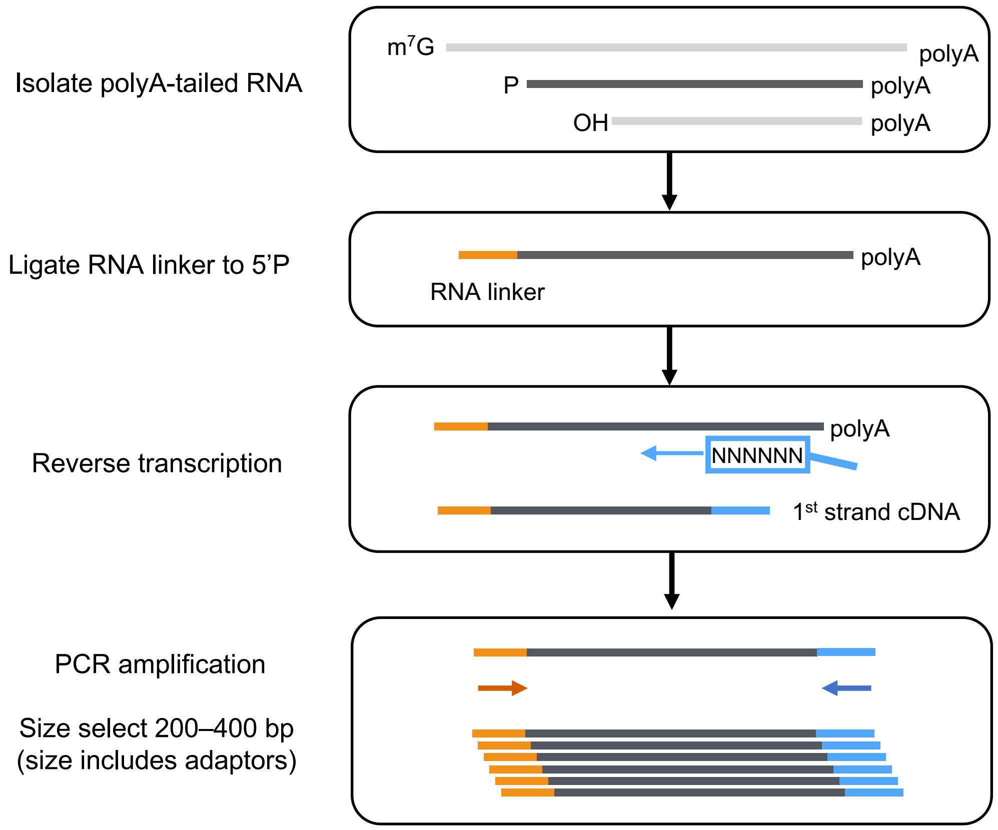

Supplementary Figure S1. Degradome-seq workflow. PolyA-tailed RNA was isolated and an RNA linker (in orange) was ligated to fragments containing a 5'phosphate, a hallmark of Drosha cleavage. All RNAs were reverse transcribed with random hexamers linked to a reverse adaptor sequence (in blue). Captured 5'phosphate-containing polyA RNAs were then PCR amplified with primers to the two adaptors, and size-selected for high-throughput sequencing on an Illumina NextSeq 500 platform.

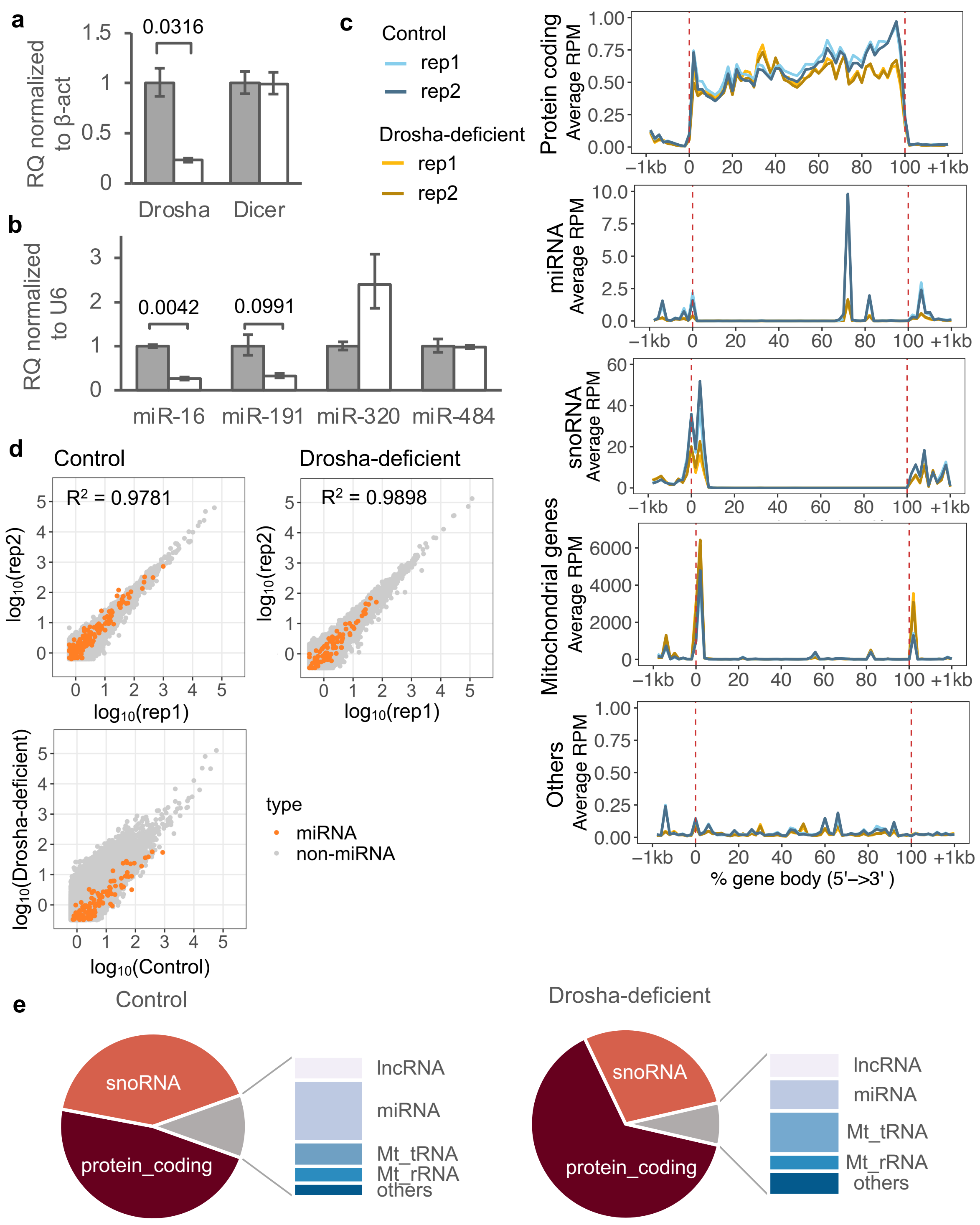

Supplementary Figure S2. Quality control for control and Drosha-deficient Degradome-seq libraries. **(a)** qRT-PCR analysis for Drosha and Dicer mRNA levels in Drosha-deficient ESCs, normalized to β-actin. **(b)** Taqman qRT-PCR analysis for expression of select mature canonical miRNAs (miR-16 and miR-191) and non-canonical miRNAs (miR-320 and miR-484) in Drosha-deficient ESCs, normalized to U6 snRNA. For **(a)** and **(b)**, bars indicate mean ± S.E.M (n = 2). Statistical testing: two-tailed independent t-test. The P-values are indicated above the bars. **(c)** Gene body coverage of Degradome-seq libraries. **(d)** Correlation between replicate degradome-seq libraries. Each dot represents the depth of the 5' end of the read at each locus. Loci within annotated miRNA gene are highlighted in orange. **(e)** Distribution of pile-up sites in control and Drosha-deficient Degradome-seq libraries.

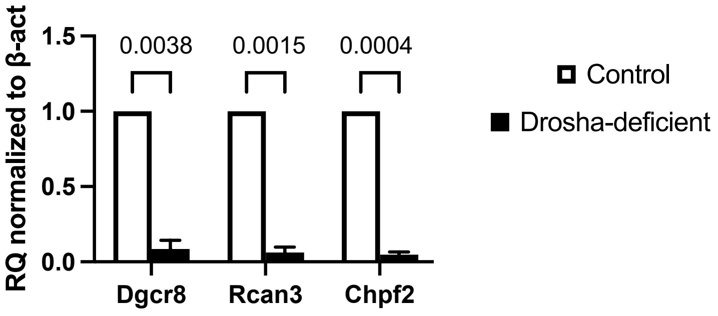

Supplementary Figure S3. Gene-specific degradome qRT-PCR analysis. Gene-specific degradome qPT-PCR analysis was performed capturing polyA RNAs and ligating a common adaptor to the residual 5' phosphate. PCR for selected Drosha targets with a reverse transcript specific primer and a forward primer in the adaptor. The means ± S.E.M (n = 2), normalized to β-actin are shown. Statistical testing: two-tailed independent t-test. The P-values are indicated above the bars.

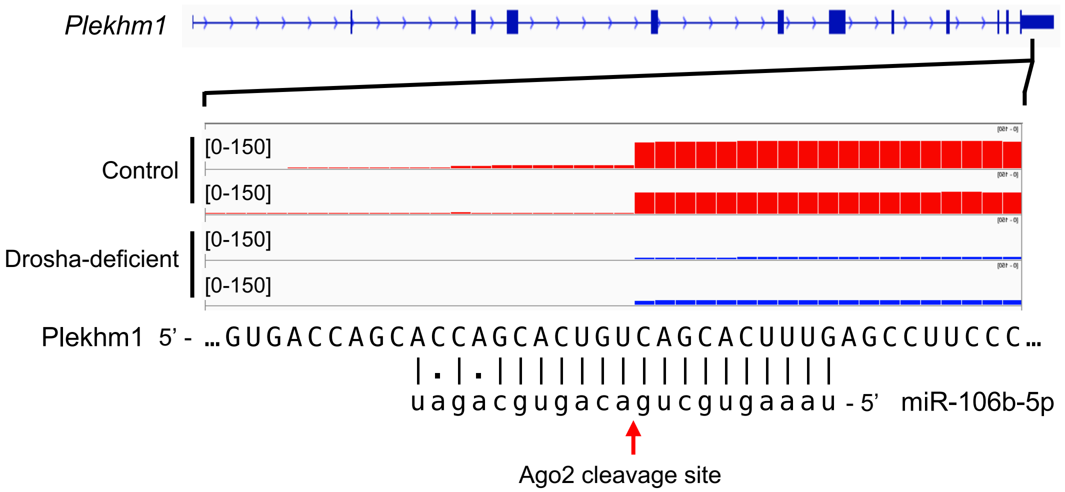

Supplementary Figure S4. Ago2-dependent miRNA-guided cleavage of Plekhm1. Shown is the Degradome-seq read pile-up in the *Plekhm1* gene comparing Drosha-deficient (blue) and control (red) ESCs. This is a known Ago2-dependent cleavage site resulting from high complimentary with miR-16-5p sequences. The read depth range is indicated in the square brackets.

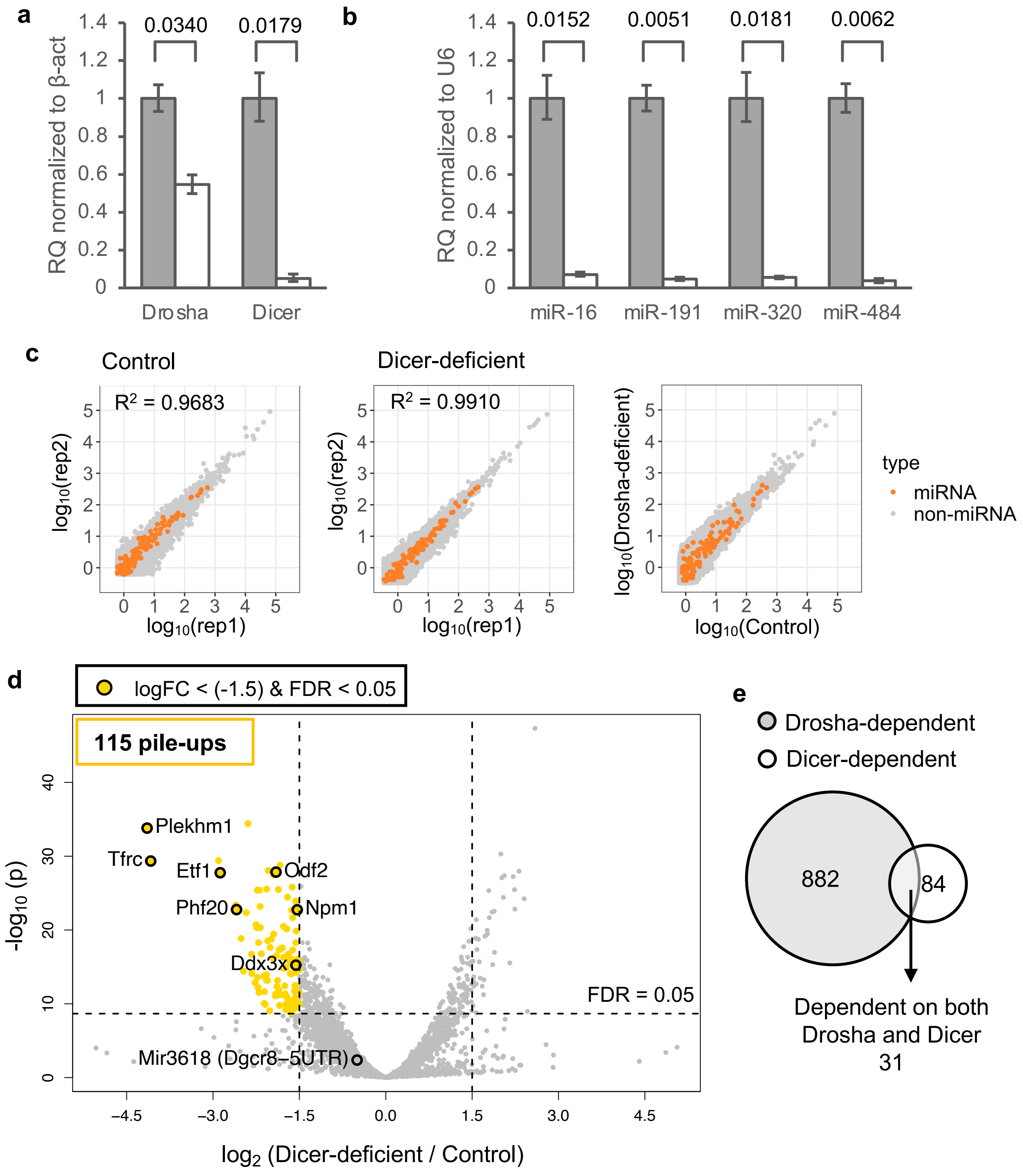

Supplementary Figure S5. Dicer-dependent Degradome-seq pile-up sites. **(a)** qRT-PCR analysis of Drosha and Dicer mRNA levels in Dicer-deficient versus control ESCs, normalized to β-actin mRNA. **(b)** Taqman qRT-PCR analysis for expression of selected mature canonical miRNAs (miR-16 and miR-191) and non-canonical miRNAs (miR-320 and miR-484) in Dicer deficient ESCs, normalized to U6 snRNA. For **(a)** and **(b)**, bars indicate mean ± S.E.M (n = 2). Statistical testing: two-tailed independent t-test. The P-values are indicated above the bars. **(c)** Correlation between replicate degradome-seq libraries. Each dot represents the depth of the 5' end of the read at each locus. Loci within annotated miRNA gene are highlighted in orange. **(d)** Volcano plot of site pile-ups comparing Dicer-deficient and control ESCs. Sites that are significantly decreased (logFC < [-1.5] and FDR <0.05, 115 sites) in Dicer deficient cells are indicated in yellow. **(e)** Venn diagram of cleavage sites in ESCs that are dependent on Drosha (decreased in Drosha-deficient cells) versus Dicer (decreased in Dicer-deficient cells).

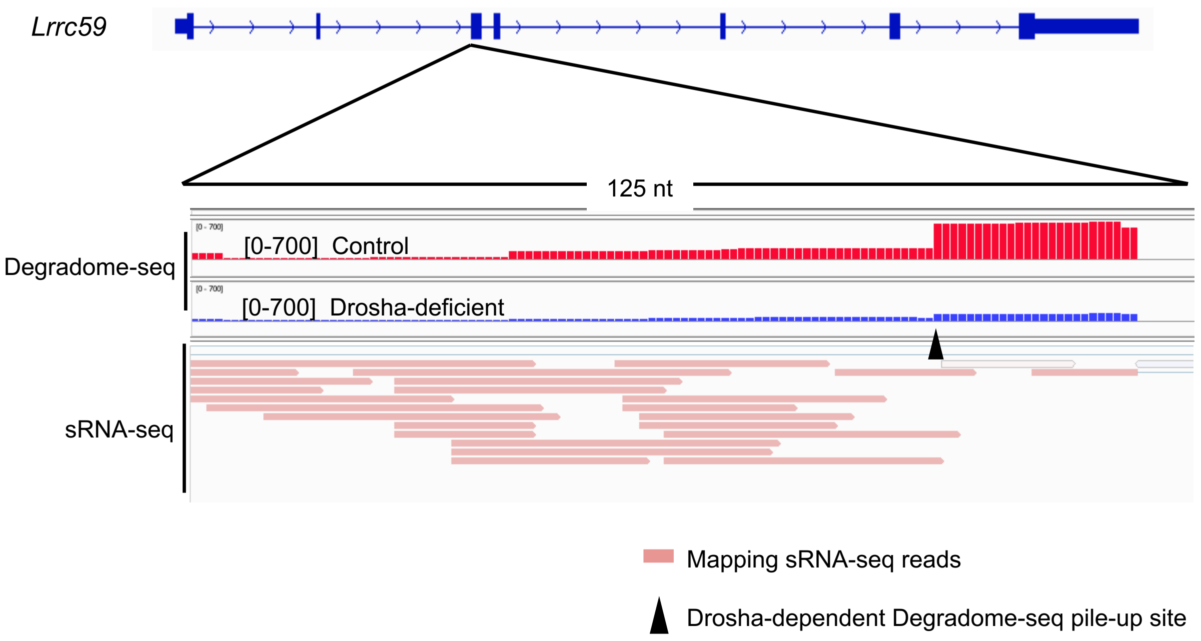

Supplementary Figure S6. Alignment of sRNA-seq reads mapping to the Drosha-dependent cleavage site in *Lrrc59* (indicated by black triangle). Depth of Degradome-seq reads comparing Drosha-deficient (blue) and control (red) ESCs is shown in the upper two tracks. The data range for each track is indicated in square brackets. The third track shows a collapsed view of sRNA-seq reads mapping to *Lrrc59*.

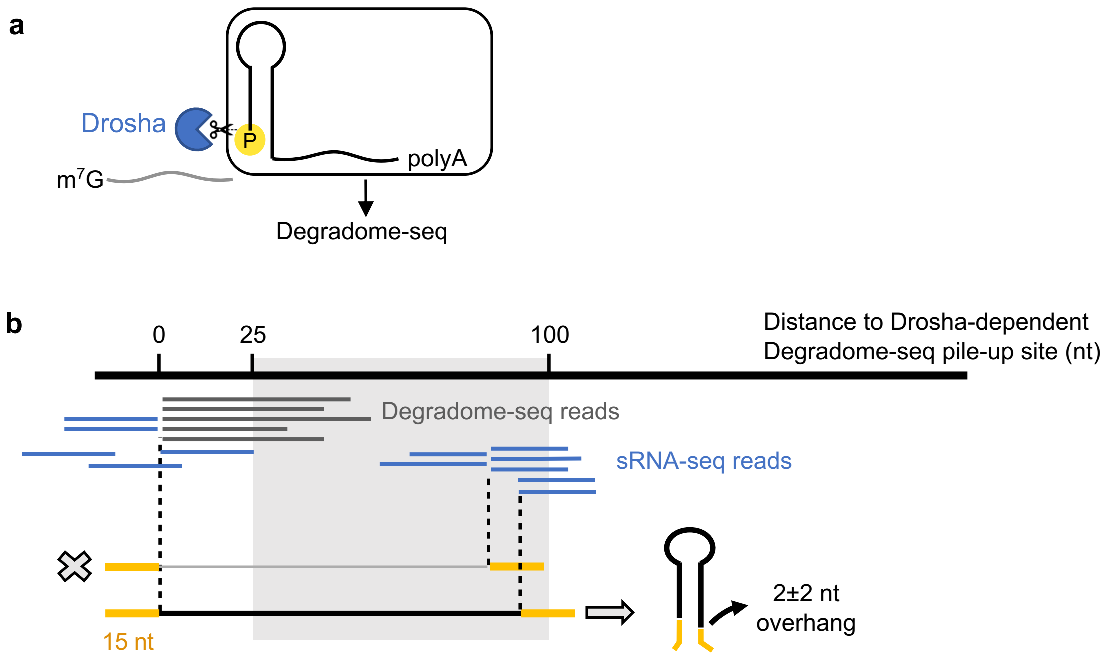

Supplementary Figure S7. Identifying the boundaries of Drosha-cleaved stem-loops that are downstream of Degradome-seq pile-up sites. **(a)** The fragment that Degradome-seq captures if Drosha only cleaves the 5' arm of the stem-loop. **(b)** Strategy for identifying Drosha-cleaved stem-loops that are downstream of the Degradome-seq pile-up site.

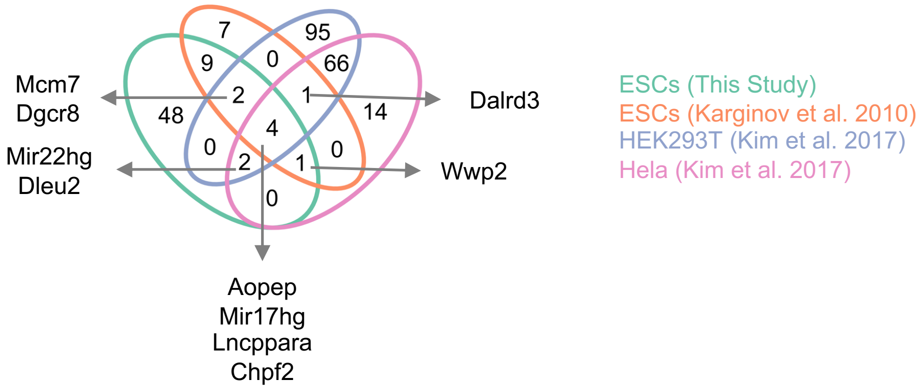

Supplementary Figure S8. Overlapping of Drosha cleavage targets in different cell types. Drosha cleavage targets identified by Karginov et al. and Kim et al. overlap with Drosha cleavage targets identified in this study. The RNAs that are identified as Drosha cleavage targets in at least three datasets are listed.

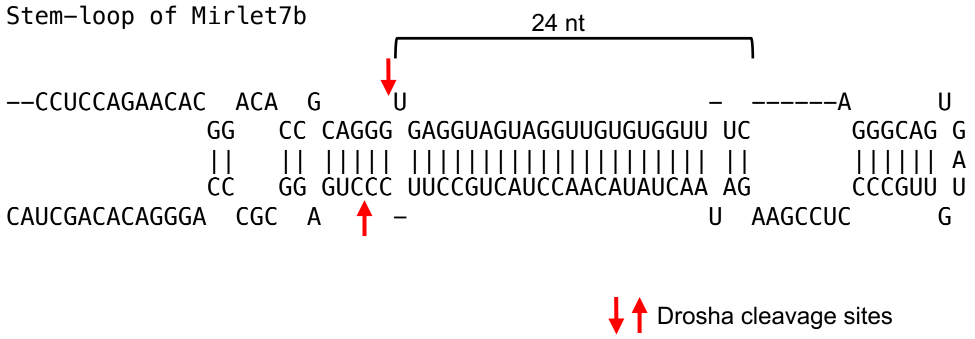

Supplementary Figure S9. The secondary structure of the stem-loop of Mirlet7b. The stem-loop has a large asymmetrical internal loop that is 24 nt away from Drosha cleavage site on the 5' arm, which serves as the terminal loop.

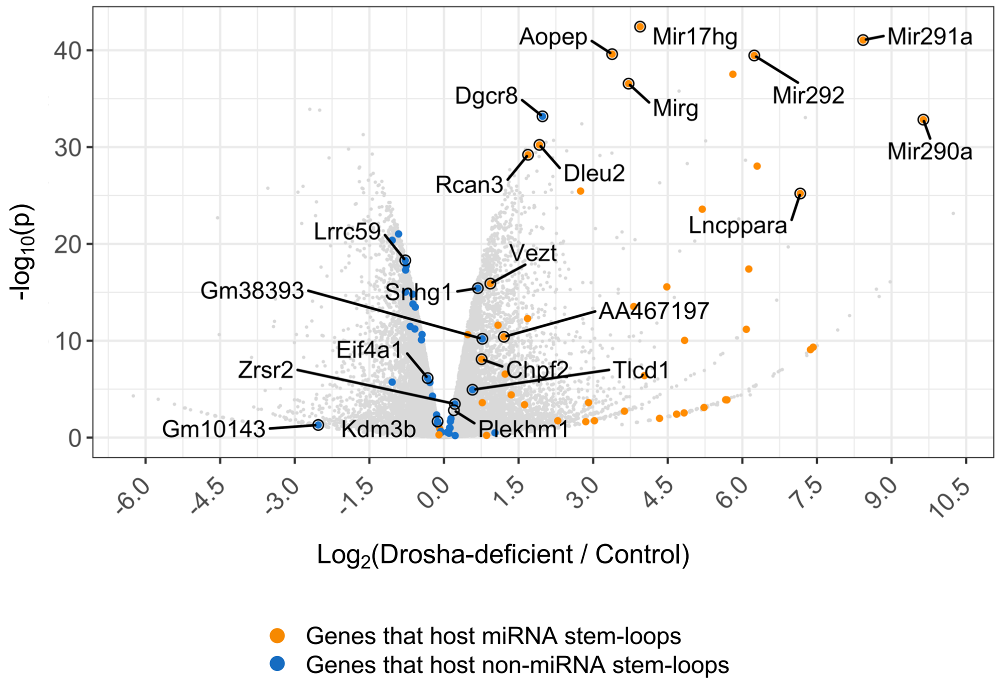

Supplementary Figure S10. Volcano plot of RNA-seq data comparing Drosha-deficient and control ESCs. Genes hosting miRNA stem-loops (orange) or non-miRNA stem-loops (blue) that are cleaved by Drosha in ESCs are highlighted.
